# Minimum design requirements for a poroelastic mimic of articular cartilage

**DOI:** 10.1016/j.jmbbm.2022.105528

**Published:** 2022-10-23

**Authors:** Wei S. Tan, Axel C. Moore, Molly M. Stevens

**Affiliations:** a Department of Materials, Department of Bioengineering and Institute of Biomedical Engineering, Imperial College London, London, UK; b Department of Biomedical Engineering, University of Delaware, Newark, DE, USA

**Keywords:** Poroelastic, Cartilage, Multiphasic mechanics, Fiber reinforced, FiHy™, Engineered cartilage

## Abstract

The exceptional functional performance of articular cartilage (load-bearing and lubrication) is attributed to its poroelastic structure and resulting interstitial fluid pressure. Despite this, there remains no engineered cartilage repair material capable of achieving physiologically relevant poroelasticity. In this work we develop in silico models to guide the design approach for poroelastic mimics of articular cartilage. We implement the constitutive models in FEBio, a PDE solver for multiphasic mechanics problems in biological and soft materials. We investigate the influence of strain rate, boundary conditions at the contact interface, and fiber modulus on the reaction force and load sharing between the solid and fluid phases. The results agree with the existing literature that when fibers are incorporated the fraction of load supported by fluid pressure is greatly amplified and increases with the fiber modulus. This result demonstrates that a stiff fibrous phase is a primary design requirement for poroelastic mimics of articular cartilage. The poroelastic model is fit to experimental stress-relaxation data from bovine and porcine cartilage to determine if sufficient design constraints have been identified. In addition, we fit experimental data from FiHy™, an engineered material which is claimed to be poroelastic. The fiber-reinforced poroelastic model was able to capture the primary physics of these materials and demonstrates that FiHy™ is beginning to approach a cartilage-like poroelastic response. We also develop a fiber-reinforced poroelastic model with a bonded interface (rigid contact) to fit stress relaxation data from an osteochondral explant and FiHy™ + bone substitute. The model fit quality is similar for both the chondral and osteochondral configurations and clearly captures the first order physics. Based on this, we propose that physiological poroelastic mimics of articular cartilage should be developed under a fiber-reinforced poroelastic framework.

## 1 Introduction

Over the last several decades there has been increasing interest in developing functional materials to replace or regenerate cartilage. Since cartilage is a load-bearing tissue that supports a low friction sliding interface, it is imperative to engineer a synthetic implant that can perform the mechanical roles of cartilage. Due to its unique composition and structure, it has been observed that, upon contact, extracellular fluid is pressurized, which stiffens the contact, reduces the load carried by the solid matrix, and provides the lowest friction coefficient. However, this same fluid pressure also drives fluid from the tissue leading to decreased pressure, increased deformation, and increased friction over time (McCutchen, 1962). These observations guided the development of biphasic-poroelastic theory for cartilage mechanics (Mow et al., 1980; Lai and Mow, 1980a; Armstrong et al., 1984; Soltz and Ateshian, 1998; Forster and Fisher, 1999; Caligaris and Ateshian, 2008; Ateshian, 2009; Biot, 1941; Basalo et al., 2004) which describes the physics leading to interstitial fluid pressurization.

Over the last four decades poroelastic theory has been expanded upon (*e.g*., poroviscoelasticity (Wilson et al., 2005a; Wilson et al., 2004a), strain dependent permeability (Lai et al., 1981), anisotropic fiber distribution (Ateshian, 2007; Ateshian et al., 2009), and zonally graded material properties (Wilson et al., 2004b, 2007)) and repeatedly shown to be an excellent predictor of the creep and stress relaxation response of cartilage in both confined and unconfined compression (Soltz and Ateshian, 1998; Basalo et al., 2004). Furthermore, experimental interrogation of cartilage mechanics has uncovered unexpected (*e.g*., migrating contacts (Caligaris and Ateshian, 2008), tribological rehydration (Moore and Burris, 2016) and regions of sub-atmospheric fluid pressures (Han and Eriten, 2018)) but predictable behavior based on poroelastic theory. Despite these advancements in understanding the natural tissue and their underpinning in poroelasticity, there remains no demonstration of a physiologically relevant poroelastic mimic for cartilage. While a few cartilage mimics have been reported to exhibit some similarities to native cartilage, none have rigorously demonstrated the ability to generate interstitial fluid pressure, which is fundamental to articular cartilage function. We propose that this gap in translation is partly due to the interdisciplinary nature of the solution (multiphasic mechanics and biomaterials design) and an unclear narrative for guiding the minimum design requirements.

In this work we use an in silico approach in the open-source finite element software FEBio (Maas et al., 2012) to develop a physiological poroelastic cartilage mechanics model. While material models of articular cartilage have become increasingly sophisticated, we aimed to develop the simplest material model for which cartilage-like performance could be observed. In this work, we define the degree of cartilage-like performance as poroelasticity and it is quantified as the fraction of the applied load supported by fluid pressure (FLS). We fit the finite element models to representative unconfined compression stress relaxation experiments on articular cartilage and FiHy™, a composite material that has been proposed to mimic articular cartilage (Moore et al., 2019). In addition to defining the minimum material model, we evaluate how variables such as strain rate, contact interface, and fiber modulus affect FLS. These results inform the material properties and experimental conditions necessary for designing and evaluating engineered cartilage mimics.

## 2 Methods

**Fig. 1:**
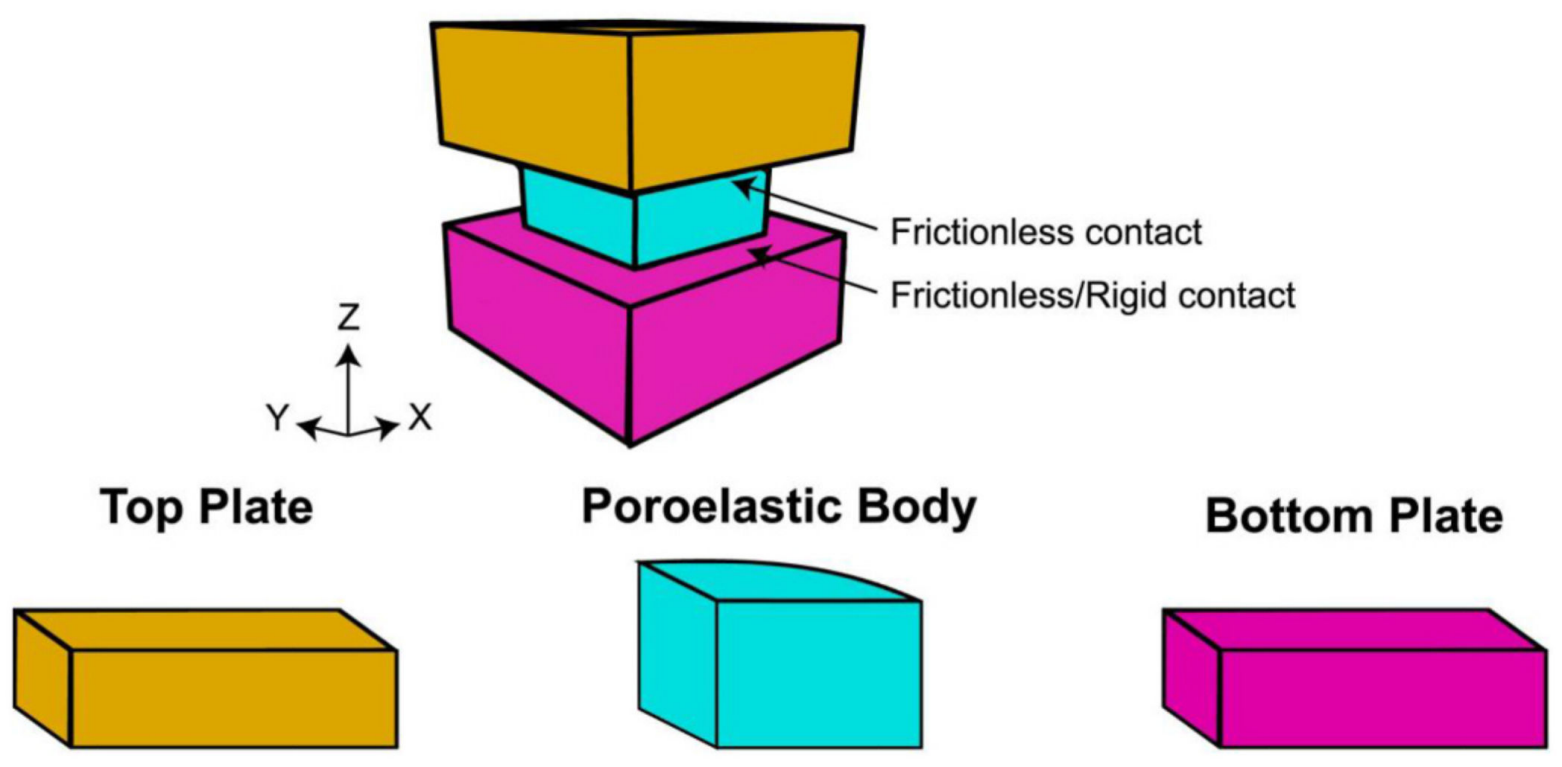
Modeled components: top plate, poroelastic body and bottom plate. The top contact is treated as frictionless, while the bottom contact is treated as frictionless or rigid depending on the question of interest. Note that all parameters of the model are defined in Cartesian coordinates.

This work leverages the framework of poroelastic theory that describes the deformation response of fluid-saturated porous media. It should be noted that in this work we do not develop new material models, but rather apply existing ones to understand the minimum design requirements. The poroelastic model is implemented in FEBio 3.4.0 (FEBio Studio 1.5), an open-source nonlinear finite element solver for mixture mechanics, fluid mechanics, reaction-diffusion, and heat transfer (Maas et al., 2012). The model geometry consists of three components: a poroelastic body, a top plate, and a bottom plate, see Fig. 1. The geometry and mesh are generated using Gmsh 4.8.4, an open-source 3D finite element mesh generator (Geuzaine and Remacle, 2009). The poroelastic body is governed by poroelastic theory, while the top and bottom plate are modeled as rigid, impermeable bodies. The rigid material model indicates that the deformation of the rigid body is zero or so small that it can be neglected. In addition, the nodal degrees of freedom for a rigid body are eliminated and replaced with the translational and rotational degrees of freedom. The contact between the top plate and the poroelastic body is modeled as frictionless, while that between the bottom plate and the poroelastic body is generally modeled as frictionless. However, we also implemented a rigid contact to investigate the role of a bonded interface in the case of an osteochondral implant.

### 2.1 Model geometry

The top and bottom plate are set up as cuboids with dimensions 5 × 5 × 2 mm, and are represented by single 8-node hexahedral (HEX8) elements each as there was no concern for deformation of the rigid bodies. The poroelastic body is set up as a quarter cylinder with radius (R) of 3 mm and height (H) of 1.5 mm, and 6302 HEX8 elements were used. We use the quarter cylinder based on symmetry conditions and the reduced computational cost. Hexahedral elements are preferred over tetrahedral elements due to the limited availability of quadratic tetrahedron elements in combination with effective contact algorithms, and the perceived increased computational cost of quadratic finite elements (Maas et al., 2016). A mesh convergence study was performed along the radial, axial, and circumferential directions to balance computational cost and accuracy, see Supplemental Information *7.1 Mesh Convergence*.

### 2.2 Poroelastic theory

Poroelastic theory (Maas et al., 2011, 2012) describes the material as a binary mixture of two constituents, namely solid (*s*) and fluid (*f*). Both constituents are assumed to be intrinsically incompressible, but the mixture can change volume when fluid enters or leaves the porous body. Under quasi-static conditions, the mixture mass balance and momentum balance reduce to

(Eq.1) $$\text{div}({\text{v}}^{\text{s}}+\text{w})=0,$$

and

(Eq.2) divσ=0,

respectively, where **v**^s^ is the solid matrix velocity, **w** is the volumetric flux of fluid relative to solid (volume of fluid passing through a crosssection of the mixture, per mixture area, and per time), and ***σ*** is the Cauchy stress for the mixture.

The relation between relative fluid flux ***w*** to fluid pressure *p* is given by

(Eq.3) w=−k⋅gradp

where ***k*** is the hydraulic permeability tensor.

Since the mixture is porous, the Cauchy stress for the mixture can be decoupled into the solid and fluid components,

(Eq.4) $$\sigma =-p\text{I}+\;\sigma^{\text{e}}$$

where **I** is the identity tensor and ***σ*_e_** is the stress resulting from solid matrix strain. Consequently, the traction force or load of the mixture (***f***) can be decoupled into two contributions as well when the stresses are integrated over the same surface, giving

(Eq.5) $$\text{f}=-{\text{f}}^{\text{f}}+{\text{f}}^{\text{e}}$$

### 2.3 Material models

The simplest model for permeability assumes isotropy and strain independence, hence the permeability tensor is given by,

(Eq.6) k=kI

where *k* is a scalar material constant.

The solid ground matrix is modeled as a compressible neo-Hookean material and its strain energy density function is given by,

(Eq.7) $$\Psi =\frac{\mu }{2}(I_{1}-3)-\mu \;\text{ln}\;J+\frac{\lambda }{2}{\left(\text{ln}\;J\right)}^{2}$$

where *μ* and *λ* are the shear modulus and first Lamé parameter from linear elasticity, and *J* is the volume ratio that describes the volume change of the solid. The shear modulus and first Lamé parameter are related to Young’s modulus (*E*) and Poisson’s ratio *ν* via $\mu =\frac{E}{2(1+\nu )}$ and $\lambda =\frac{E\nu }{\left(1+\nu )(1-2\nu \right)}$. *I*_1_ denotes the first invariant (trace) of the right Cauchy-Green deformation tensor ***C***. Therefore, only two material parameters (*E* and *ν*) are needed to describe the Neo-Hookean material. This model is referred to as the poroelastic model in the following sections.

A solid mixture model that incorporates continuous fibers (*fi*) within a neo-Hookean ground matrix (*g*) is used to model the effect of fiber reinforcement on the poroelastic response. This model assumes that the strain energy density of the mixture is the summation of the strain energy densities of all the constituents. Assuming no explicit dependencies between the fibers and ground matrix, and no residual stresses in the solid mixture, the strain energy density function of the mixture reduces to

(Eq.8) $$\Psi =\sum_{\alpha =\text{fi},\text{g}}\Psi^{\alpha }(\text{F},\rho^{\alpha })$$

where **F** denotes the deformation gradient and *ρ^α^* the apparent density of constituent *α*, and the Cauchy stress for the solid mixture becomes

(Eq.9) $$\sigma^{\text{e}}=\sum_{\alpha =\text{fi},\text{g}}\sigma^{\alpha }$$

In other words, the stress in the solid phase can be evaluated simply by summing the stresses in the fibers and ground matrix.

To implement this model, the fibers are modeled as a continuous fiber distribution, of which the strain energy density function integrates the contributions from fiber bundles oriented along all directions emanating from a point in the continuum,

(Eq.10) $$\Psi (\text{C})=\int_{A}H\;(I_{n}-1)\;\frac{1}{2\pi }\Psi_{n}\;(I_{n})\;dA$$

where *A* represents a unit circle over which the integration is performed, *I_n_* = **n⋅C⋅n** denotes the normal component of **C** along **n** (the square of the stretch ratio along that direction), and ***n*** is the unit vector along the fibre orientation in the reference configuration. *H*(**·**) is the Heaviside unit step function that includes only fibers that are in tension. The fiber density distribution is described using a circular distribution which models a transversely isotropic 2D distribution.

Meanwhile, *Ψ_n_* represents the strain energy density of the fiber bundle oriented along ***n***. The fiber bundles are modeled using an exponential power law and the strain energy density function is given as

(Eq.11) $$\Psi_{n}(I_{n})=\frac{\xi }{\alpha \beta }(\text{exp}\;[\alpha (I_{n}-1{)}^{\beta }]-1)$$

Overall, three material parameters (*ξ*, *α* and *β*) are needed to describe the continuous fiber distribution model. This fiber-reinforced poroelastic model is referred to as the fiber-reinforced model in the following sections.

### 2.4 Boundary conditions

**Fig. 2:**
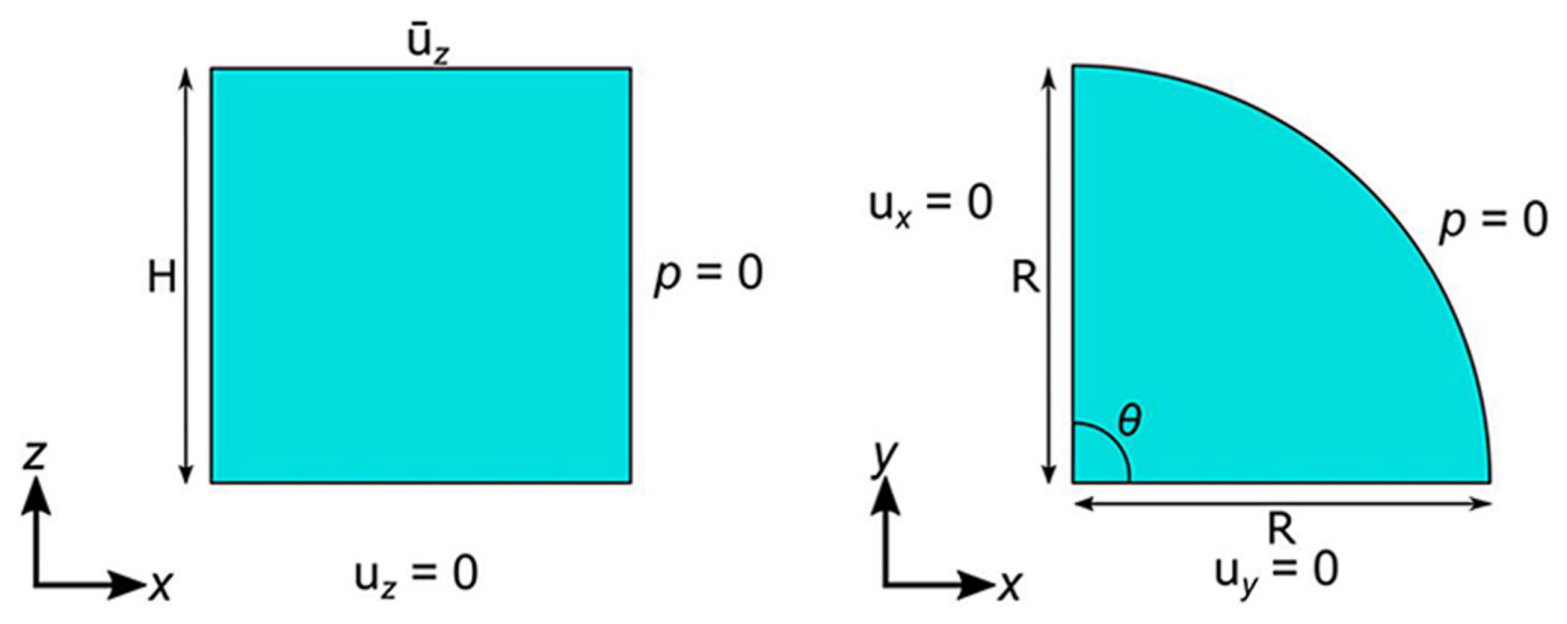
2D visualization of the boundary conditions on the poroelastic body for the case where the bottom contact is frictionless in the front view (left) and the top view (right).

The following boundary conditions are applied to the different surfaces (see Fig. 2 and Table 1),

$$\begin{array}{l} {\text{u}}_{\text{x}}\;(0,\;\text{y},\;\text{z},\;\text{t})=0\\ {\text{u}}_{\text{y}}\;(\text{x},\;0,\;\text{z},\;\text{t})=0\\ \text{p}\;(R\;\text{cos}\;\theta ,\;R\;\text{sin}\;\theta ,\;\text{z},\text{t})=0\;for\;0^{\circ }\leq \theta \leq 90^{\circ } \end{array}$$

The first two boundary conditions are Dirichlet boundary conditions where the x- and y-displacement, at *x* = 0 and *y* = 0 respectively, are fixed at all times. The third boundary condition indicates that the fluid pressure at the curved surface of the poroelastic body is zero, hence fluid can flow out of the body from this surface.

Furthermore, the stress-relaxation experiment requires the sample to be compressed between two plates using displacement-control. To model this experimental setup, a rigid displacement ū_*z*_ is prescribed onto the top plate in the negative z-direction (compression). Specifically, a linear ramp (1% strain/s unless otherwise stated) to the target displacement (10% strain) is applied and maintained until the end of the simulation. The 10% strain was chosen to model in vivo cartilage strains which are typically 5–10% during daily activity (Sanchez-Adams et al., 2014; Widmyer et al., 2013; Coleman et al., 2013). Since deformation of the rigid body is negligible, the prescribed displacement is applied to the poroelastic body. Other than the prescribed rigid displacement, the top plate is constrained in the x- and y-displacement, and rotation in all three directions. The bottom plate is constrained in all displacements and rotations.

In the case of a frictionless bottom contact, the bottom surface of the poroelastic body (at z = 0) can slide in the x- and y-direction, but not in the z-direction, giving

$${\text{u}}_{\text{z}}\;(\text{x},\;\text{y},\;0,\;\text{t})=0$$

However, to model for a fully bonded contact, the nodes of the deformable poroelastic body in contact with the bottom plate are coupled to the rigid body (implemented as rigid contact). Essentially, the bottom surface of the poroelastic body is constrained in all directions and is not allowed to slide freely as in the case of a frictionless contact, *i*. *e*.

$$\begin{array}{l} {\text{u}}_{\text{x}}\;(\text{x},\;\text{y},\;0,\;\text{t})=0\\ {\text{u}}_{\text{y}}\;(\text{x},\;\text{y},\;0,\;\text{t})=0\\ {\text{u}}_{\text{z}}\;(\text{x},\;\text{y},\;0,\;\text{t})=0 \end{array}$$

Moreover, the top and bottom plates are impermeable, giving rise to the following boundary conditions:

$${\text{w}}_{\text{z}}\;(\text{x},\;\text{y},\;\text{H},\;\text{t})=-{\text{w}}_{\text{z}}\;(\text{x},\;\text{y},\;0,\;\text{t})=0$$

### 2.5 Stress relaxation simulation & FLS quantification

**Fig. 3:**
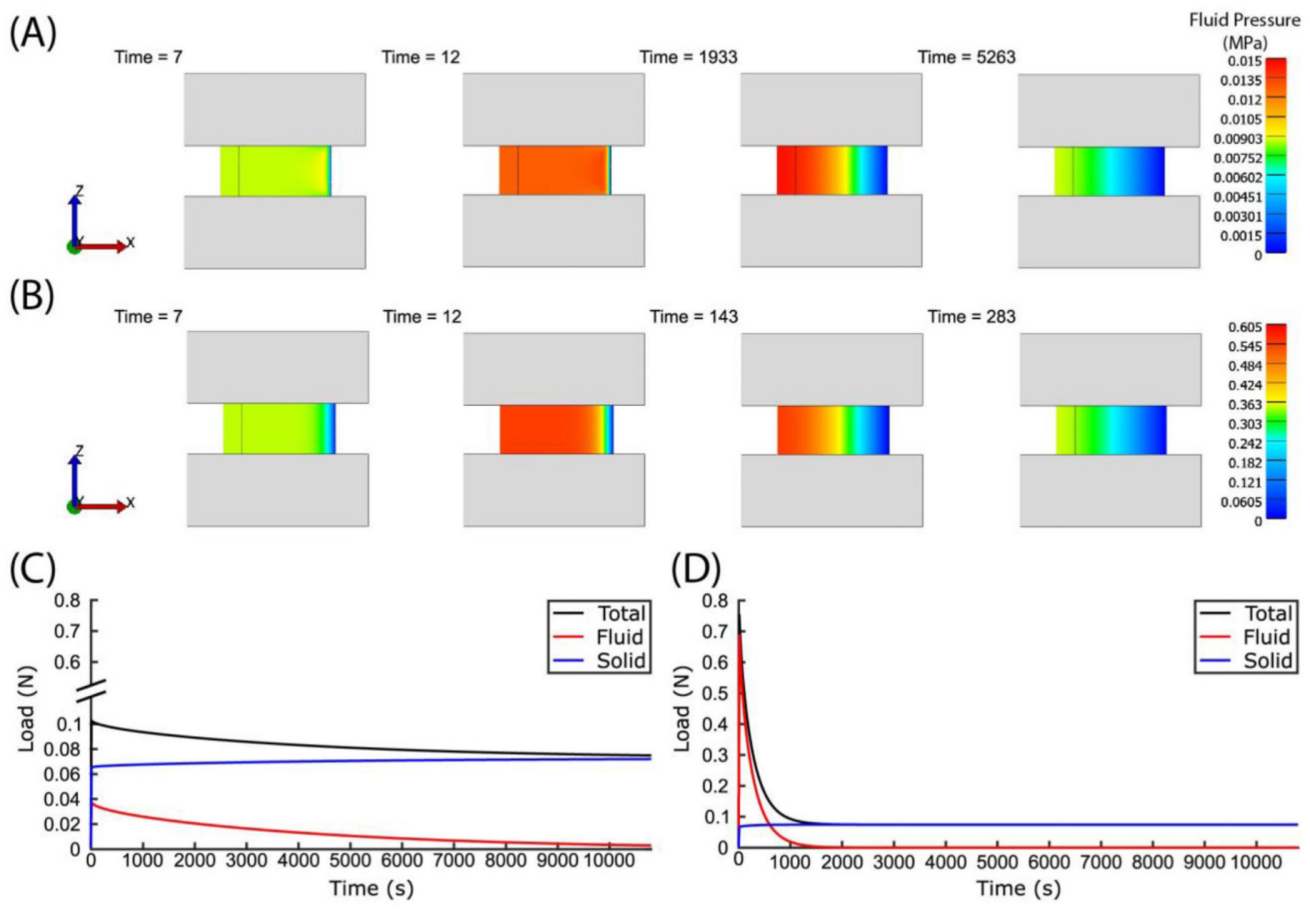
(A, B) Effective fluid pressure distribution within the (A) poroelastic model and (B) fiber-reinforced model (front view) at various times in the simulation. Initially, fluid pressure builds to support the applied compressive load, but as time goes on, fluid flows out of the body and the fluid pressure subsides. Note that the colors correspond to different fluid pressures in (A) and (B), and the time steps for (A) and (B) are different. (C, D) Plot of load (evaluated at the top surface of the (C) poroelastic model and (D) fiber-reinforced model) against time. The total applied load is decoupled into two contributions: solid and fluid. All loads are given as positive values. (A) and (C) are obtained from the poroelastic model, while (B) and (D) are obtained from the fiber-reinforced model using the same initial conditions.

An example stress relaxation simulation is shown in Fig. 3. There are several noteworthy observations. First, fluid pressure decays with time due to the permeability of the poroelastic body. Second, fluid pressure is greatest at the center of the poroelastic body for most times as it is the furthest from the zero-pressure boundary. Third, in an isotropic frictionless contact the fluid pressure is uniform along z.

Based on mixture theory, the load is partitioned between the fluid and solid matrix of the poroelastic body, and the sum of their contributions equals the total applied load (Fig. 3C and D). The fluid load support (FLS) of the poroelastic body is simply the ratio of the fluid load to the total applied load. To evaluate the FLS on a particular surface on the poroelastic body, the fluid load and the total load on that surface need to be calculated and can be found by integrating the fluid pressure and the minimum principal stress (stress normal to the poroelastic body) over that surface respectively, giving

$$FLS=|\frac{\int_{\text{S}}\text{p}\;\text{da}}{\int_{\text{S}}\sigma_{\text{p},\text{min}}\;\text{da}}|$$

where *S* is the surface of interest, and σ_p,min_ is the minimum principal stress. To gauge the transient response of the poroelastic body, we calculate the time for FLS to fall below 0.5%. The integrations and thresholding are performed using custom written MATLAB® (R2019b) scripts.

### 2.6 Model parameters

The work presented here includes one material coefficient (*k*) to describe the permeability of the poroelastic body, and two elasticity parameters (*E* and *ν*) to describe the ground matrix for the poroelastic model. In the fiber-reinforced model, three additional elastic parameters are used to describe the fibers (*ξ*, *α* and *β*). Table 2 provides a summary of the material parameters that are used within the simulation.

The fluid volume fraction *φ*_f_ of articular cartilage generally ranges between 0.7 and 0.9 and variations over this range have negligible influence on the predicted response (Ateshian et al., 2009), hence *φ*^f^ is simply chosen to be 0.8. This value also corresponds to the experimentally measured fluid volume fraction of FiHy™. The values for *k*, *E*, and *ν* are informed by past theoretical and experimental studies of articular cartilage, where *k* is reported to be on the order of 10^–3^ mm^4^/N · s (Ateshian, 2009), The Young's modulus is approximately 0.5 MPa (Park et al., 2004; Armstrong and Mow, 1982), and the drained Poisson's ratio is between 0.02 and 0.16 (Ateshian et al., 2004), which is also in agreement with the experimentally measured Poisson's ratio for FiHy™.

To date, few published studies have implemented the constitutive model for continuous fiber distribution. Therefore, our estimations of the fiber parameters *ξ*, *α*, and *β* are guided by conventions amongst the FEBio community. *ξ* is often chosen to be in the range of 1–4 MPa in models for continuous fiber distribution, ellipsoidal fiber distribution, spherical fiber distribution and discrete fiber bundles (Ateshian et al., 2009; FEBio Forum, 2021; Wilson et al., 2005b). *α* is chosen to be 0 to provide the power-law relation for each fiber bundle as this relation has been validated for cartilage by Chahine et al. (2004). *β* is set to 2 so that it produces a piecewise-linear stress-strain response across the origin (Ateshian et al., 2009).

### 2.7 Model fitting

In this work, we use a manual fitting approach for purposes of speed and global fitting to the data. Using the built-in parameter optimization provides the best fit by minimizing the residual; however, this results in a fit that is biased toward zero fluid pressure, and thus fails to capture the initial response of the system. Using an iterative manual fitting approach we are better able to fit the initial response without the need for weighting functions.

We use the following process to manually fit the data.

1. Fit the equilibrium response, which is almost entirely controlled by *E*.
2. Fit the peak force, which is controlled by *E* and *ξ.* Since *E* is fixed in step 1, only *ξ* requires iteration.
3. Fit the transient response which is controlled by *ξ* and *k*. Since *ξ* is fixed in step 2, only *k* needs to be adjusted.

The experimental data of the chondral specimens (bovine cartilage, porcine cartilage and FiHy™) are fitted with the fiber-reinforced model using a frictionless top and bottom contact. For the osteochondral specimens (porcine osteochondral explant and FiHy™ + bone substitute), a fiber-reinforced model with a fully bonded bottom contact is implemented. The bonded contact is used to more faithfully reproduce the true boundary conditions for osteochondral specimens and the fused FiHy™ + bone substitute. The geometry of the poroelastic body is adjusted according to the actual specimen geometry (see Table 3).

To assess the quality of fit, the standard error of regression S is calculated.

$S=\sqrt{\frac{SS_{res}}{n-2}}$ where $SS_{res}=\sum_{i}{\left(y_{i}-f(x_{i})\right)}^{2}$. x_i_ and *y_i_* are the experimental time and force data, f(x_i_) is the predicted value obtained from the simulation model and n is the total number of data points. A good fit should have S values approaching 0.

### 2.8 Experimental method

Cylindrical biopsies of bovine articular cartilage, porcine articular cartilage, and FiHy™ were tested in stress relaxation and fit by the poroelastic models. We extracted osteochondral cores from stifle joints on the day of butchering and froze the explants at –20 °C until the day of testing. Frozen specimens were thawed under ambient conditions and submerged in fresh 1X phosphate buffered saline (PBS) for 2 h prior to cartilage isolation or testing. We isolated articular cartilage from the osteochondral cores using a biopsy punch and cut along the cartilagebone interface. The cartilage cylinder was then placed in a V-block to cut the bottom surface into a right cylinder. The articular cartilage surface remained intact. For osteochondral testing the explant remained intact. FiHy™ specimens were provided as a sheet in both a cartilage-only and cartilage + bone substitute format. 6 mm diameter biopsy punches were used to extract FiHy™ cores for testing. Scaffolds were submerged in 1 ml of PBS for at least 2 h prior to testing.

Hydrated specimen thickness and diameter were measured in triplicate and then loaded in compression between parallel stainless-steel plates on a mechanical test bench (TA Electroforce 5500). 100 μl of PBS was placed directly on the sample and a humidity chamber was placed around the entire setup to ensure hydration throughout testing. A 22.2 N tension-compression load cell was used to quantify the compressive force while an internal displacement sensor recorded the axial position.

The following stress-relaxation protocol was implemented: (1) ramp toward the sample at 0.5 V/s (~0.6 mm/s) until reaching the target load, (2) hold the position in which the target load was achieved until equilibrium, and (3) unload the specimen. The target peak compressive stress was 0.4 and 0.2 MPa for articular cartilage and FiHy™ respectively. The time for equilibration was based on prior knowledge of the specimen’s relaxation behavior. Note that voltage control was used when loading the sample as it offered the highest loading rates and less stability issues in the control algorithm.

## 3 Results and discussion

### 3.1 Effect of loading rate on the transient response

**Fig. 4:**
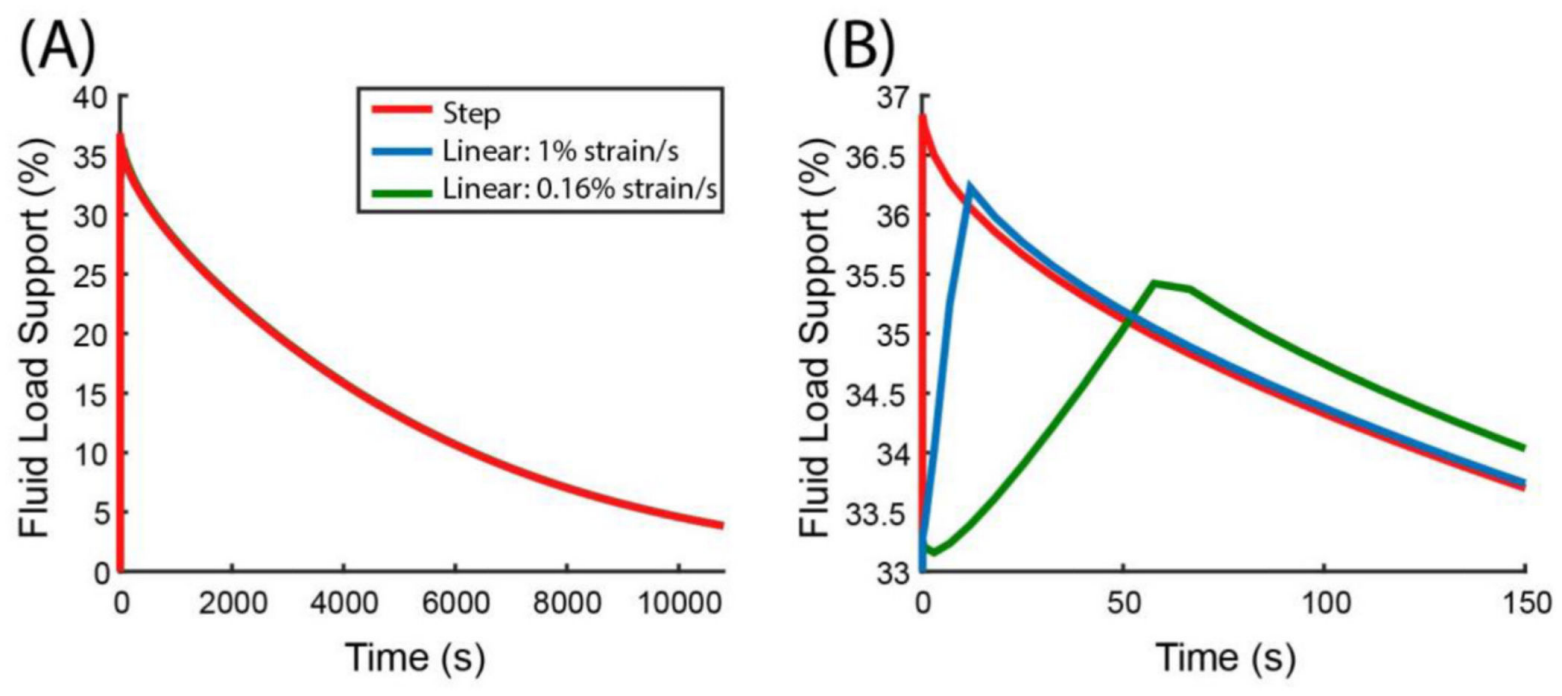
The effect of strain rate on FLS. (A) The response for the entire simulation period. (B) The early time response for FLS. A step displacement condition can be thought of as an infinitely large linear ramp rate. Note that the result shown here is obtained using the poroelastic model without fiber-reinforcement.

We observed very little effect of strain rate on FLS when considering the entire simulation period (Fig. 4). In fact, its effect is only apparent in the very early period of the simulation; the difference between FLS of each case was less than 0.8% after 300 s. The peak FLS increases as the loading rate increases because the poroelastic body deforms faster than the fluid can flow out; hence it acts as if it is ‘trapped’ within the solid matrix, building fluid pressure. However, once at the target displacement the higher fluid pressure leads to an initially greater exudation rate and the FLS converges with the slower loading rates after a period of time.

Although there have not been many reports on the physiological strain rate or loading rate for the human knee due to the difficulty in obtaining reliable measurements, it is postulated by Oloyede and coworkers that the normal strain rate is about 0.5%/s (Oloyede et al., 1992), while Chia and Hull have suggested walking to produce a strain rate of 32%/s (Chia and Hull, 2008). Therefore, the values considered in this work produce an upper (step loading) and lower (0.16%/s) bound for physiological strain rates in the human knee. Since the linear ramp at 1%/s is within the suggested physiological range we have chosen it for all other investigations in this work.

### 3.2 Effect of fiber-reinforcement

**Fig. 5:**
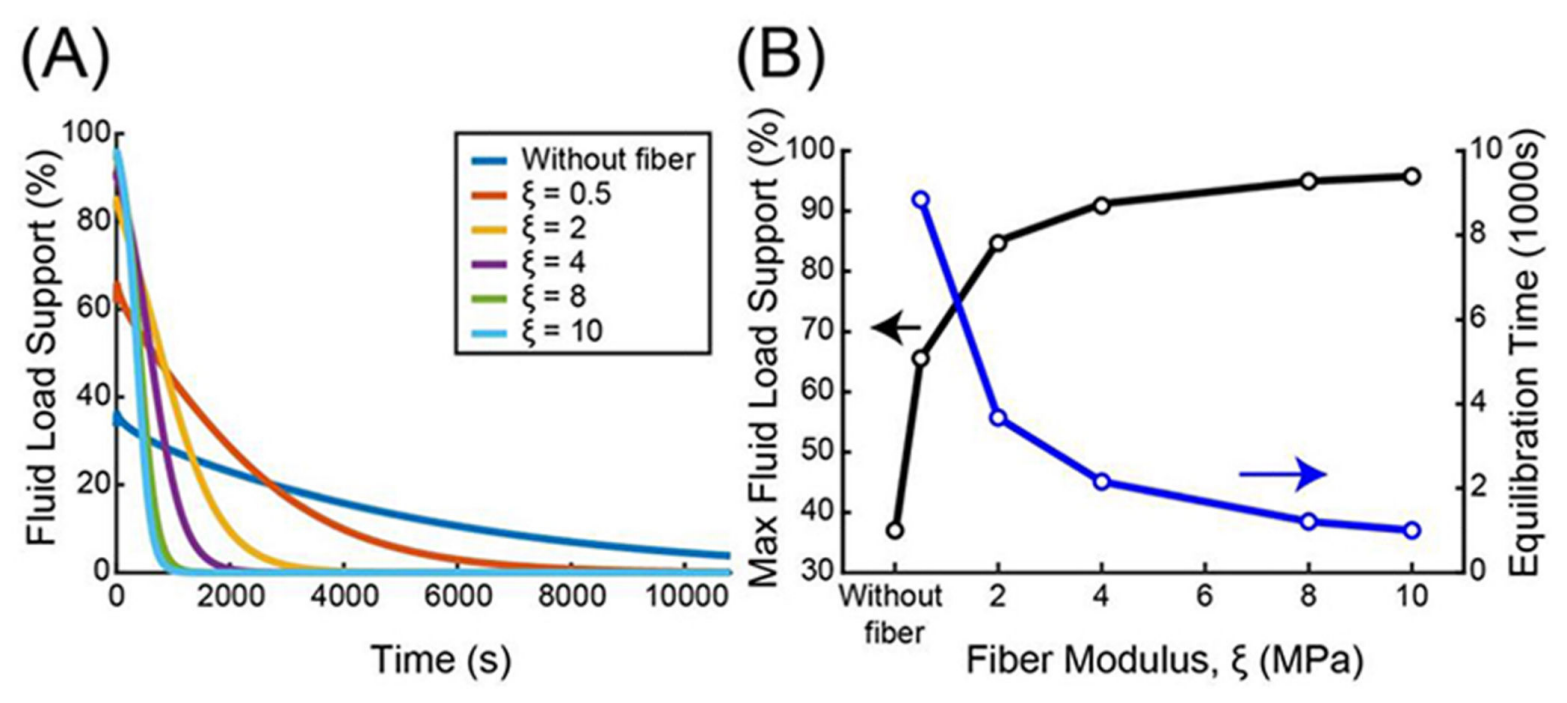
(A) Plot of FLS as a function of time for different fiber moduli *ξ*. The poroelastic model without fiber incorporation is denoted as ‘without fiber’. Note that the FLS is evaluated at the top surface of the poroelastic body. (B) The effect of fiber modulus on the maximum (peak) FLS and the time to reach equilibrium.

Tension-compression nonlinearity is one of the major advancements in modeling articular cartilage as it explains the high FLS (as high as 99%) observed experimentally (Park et al., 2003). The tension-compression nonlinearity of cartilage has been primarily attributed to the incorporation of fibers in a poroelastic material (Basalo et al., 2004; Ateshian et al., 2009; Soulhat et al., 1999), and highlights the significance of collagen fibers in articular cartilage. In the unconfined compression setup, the fibers resist radial deformation due to their high tensile modulus and provide the reaction force for hydrostatic pressure. With stiffer fibers, more radial deformation is resisted during compression and thus a higher fluid pressure can be achieved within the poroelastic body. Indeed, our simulations confirm that incorporating stiffer fibers in the poroelastic body resulted in fluid pressures that were able to support up to 96% of the total load (Fig. 5a). Our results are corroborated by the experimental findings of Basalo et al. (2004).

Interestingly, we observed that the time to reach equilibrium decreases as fiber modulus increases (Fig. 5b). This transient response of fluid pressure in a poroelastic material is characterised by its time constant (*τ*). The analytical solution to 1D permeation is (Mow et al., 1980; Armstrong et al., 1984; Ateshian, 2017),

(Eq.12) $$\tau =\frac{{\text{h}}^{2}}{{\text{H}}_{+\text{A}}\text{k}}$$

where *h* is the characteristic path length in which fluid predominantly flows (in this work, radius of the poroelastic body) and *H_+A_* = *H_A_*+ *ξ* is the combined stiffness of the Neo-Hookean ground matrix and fibers. *H_A_* = *λ* + 2*μ* is the aggregate modulus of the ground matrix and can be related to the first Lamé parameter *λ* and shear modulus *μ*. The above relationship has been repeatedly demonstrated in the literature and includes the work of Carter et al. (2007). From Eq. (12), the characteristic time constant is shown to be inversely related to the fiber modulus if all other parameters remain the same. Thus, stiffer fibers produce greater fluid pressures that create a driving force for fluid to flow out of the body, thereby reducing the time to reach equilibrium.

### 3.3 Effect of the bonded interface

Two contact interfaces, namely the frictionless contact and the fully bonded contact, are investigated in this work. The fully bonded contact is relevant to the native tissue and osteochondral implants such as FiHy™, which involve a cartilage mimic bonded to a subchondral bone substitute. In a typical stress relaxation experiment in unconfined compression, FiHy™ is compressed between two frictionless plates. This means that FiHy™ is allowed to expand freely in the radial direction, hence the top and bottom contacts can be modeled as frictionless. However, with an attached bone substitute, the bottom surface of FiHy is unable to move as freely in the radial direction. In this case, the bottom contact can be modeled as a sliding interface with an extremely large friction coefficient, or simply as a rigid contact. The implementation of a rigid contact is less computationally intensive and provides a similar result as that of the sliding interface with a large friction coefficient (see Fig S7.2), hence a rigid contact is chosen in this work. It is noted that this contact condition is realistic under the assumption that the bond between FiHy and the bone substitute is extremely strong such that no slip will occur at the interface.

**Fig. 6:**
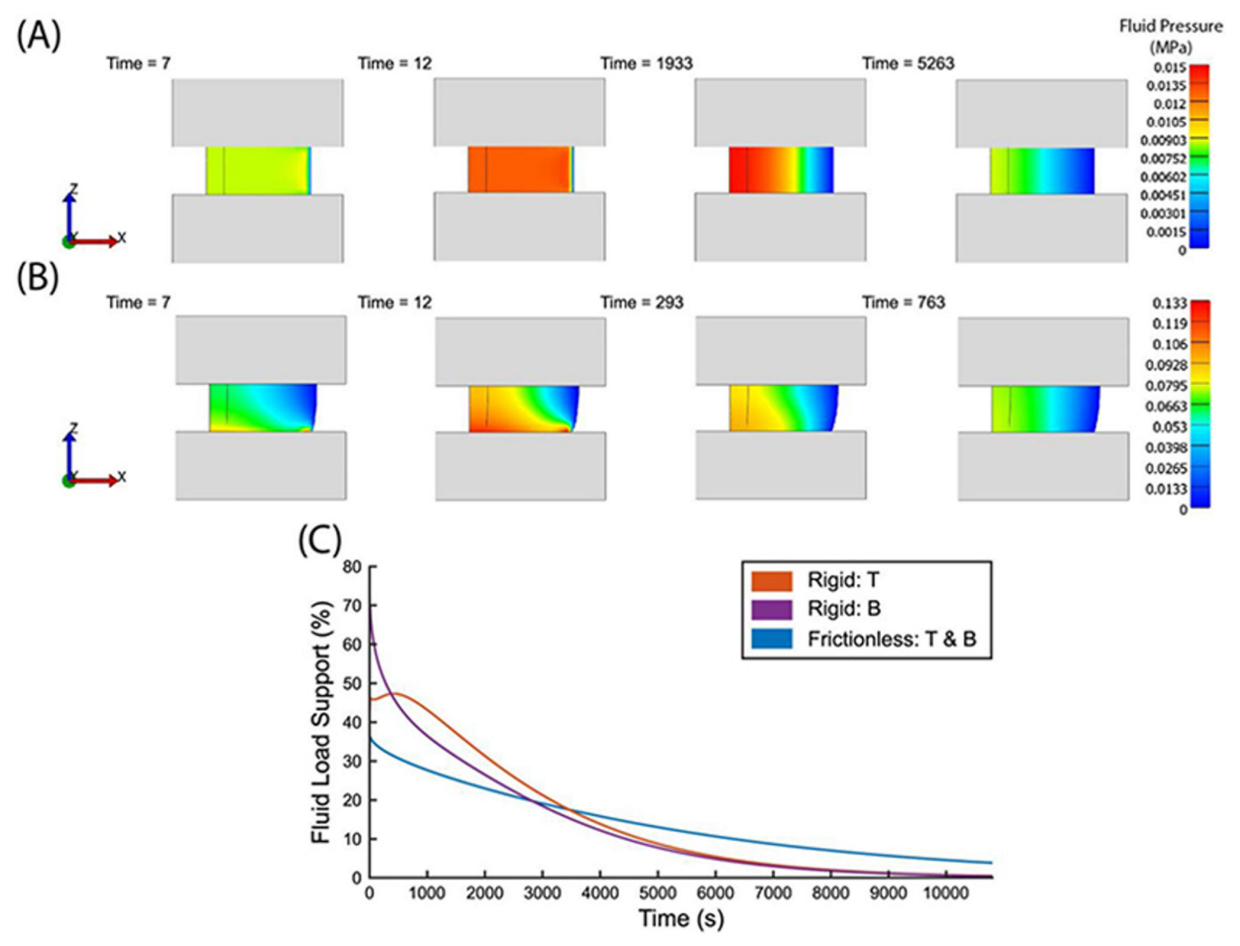
(A, B) Effective fluid pressure distribution within the poroelastic body (front view) at selected times in the simulation. (A) and (B) are obtained from the poroelastic model with (A) frictionless bottom contact and (B) fully bonded contact. Note that the scales and time steps are different for (A) and (B). (C) Plot of FLS as a function of time. The FLS is evaluated at the top (denoted as T) and bottom (denoted as B) surface of the poroelastic body.

It is important to emphasise that the effective fluid pressure, and hence the fluid load, is always uniformly distributed in the radial direction for an isotropic poroelastic body with frictionless contact. In other words, there is no axial dependence of fluid pressure and FLS is consistent for the top and bottom surface of the poroelastic body (Fig. 6C); this is not the case for the fully bonded contact. There are three important observations that can be made using a fully bonded contact: (1) the peak FLS evaluated at the top surface of the poroelastic body is higher and propagates slower when a fully bonded contact is implemented; (2) the FLS evaluated at the bottom surface (fully bonded contact) is higher than that at the top surface, but eventually equilibrates at ~5000 s for the modeled conditions; (3) the overall FLS for the top and bottom surface in the case for the rigid contact is higher than that for the frictionless contact until ~3000 s. The bonded interface creates an effective radial confinement at the bottom, and in a way acts as further fiber reinforcement. These results demonstrate that the additional constraints imposed by the rigid contact result in higher peak FLS that may have important physiological implications.

While these findings demonstrate a possible method of amplifying FLS and a mechanism nature may already exploit, there are currently no experimental findings that can validate this observation. In fact, native cartilage tissues have been shown to have higher FLS at the superficial zone than at the deep zone (near the bone); however, this is generally linked to the collagen fiber orientation (Park et al., 2003). Some studies on contact mechanics of articular cartilage have modeled the cartilage to be ideally bonded to bone (similar to the rigid contact in this work) (Wu et al., 1997; Ateshian et al., 1994), but did not focus on the relationship between contact conditions and the resulting FLS. In future work we aim to experimentally validate the role of the bonded interface on the FLS.

### 3.4 Cartilage and FiHy™

Numerous studies have demonstrated that poroelastic theory and its extensions can indeed be used to describe cartilage mechanics (Forster and Fisher, 1999; Caligaris and Ateshian, 2008; Ateshian, 2009; Soltz and Ateshian, 2000). To develop a synthetic implant that matches the mechanical properties of cartilage, it is necessary to show that the mechanical response of the implant can be described using poroelastic theory as well. Hence, the simulation models developed in this work are fit to experimental stress-relaxation data of a bovine cartilage specimen, a porcine cartilage specimen, and a representative FiHy™ sample.

**Fig. 7:**
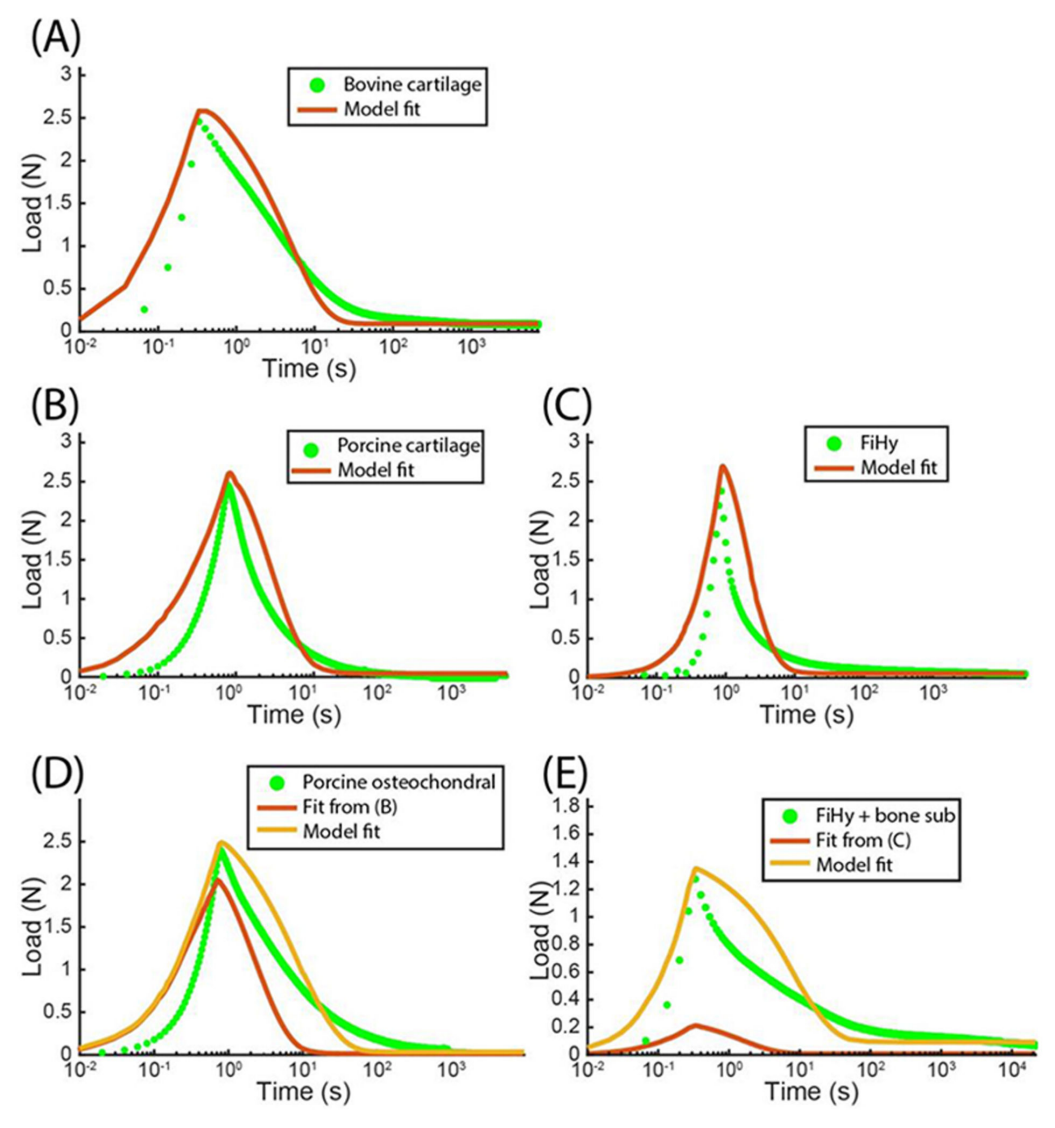
Experimental unconfined compression stress-relaxation and corresponding curve-fits for (A) bovine cartilage, (B) porcine cartilage, (C) FiHy™, (D) porcine osteochondral explant, and (E) FiHy™ attached to a bone substitute. In addition to the optimized curve fits in (D) and (E), the experimental data is also fit with parameters that were determined from their chondral counterpart in (B) and (C). (A), (B), and (C) were fit with the fiber reinforced poroe-lastic model while (D) and (E) were fit with the fiber reinforced poroelastic + rigid contact model.

As seen in Fig. 7(A-C) and Table 4, the fiber-reinforced model showed good agreement (*S* = 0.12 and 0.42) with the experimental data of bovine and porcine cartilage. The goodness of fit is strongly dependent on the inclusion of tension-compression nonlinearity, which agrees with previously published work for unconfined compression of articular cartilage (Soulhat et al., 1999; Soltz and Ateshian, 2000; Cohen et al., 1998). In comparison, the fiber-reinforced model had a slighly lower fit quality to FiHy™ (*S* = 0.47). This may be due to additional material properties that are not captured in the fiber-reinforced model, such as poroviscoelasticity or directional permeability. Despite this, the fiber-reinforced model gave a similar overall response as the experimental data and gives confidence that FiHy™ is at least partially governed by poroelasticity. The loading rates used in this study are more than an order of magnitude faster than most of the poroelastic literature using this same contact configuration (Park et al., 2003; Soltz and Ateshian, 2000; Cohen et al., 1998). We chose to use high loading rates in this work to better approximate the dynamic loading of joints. One study that looked at similar loading rates found similarly low overall agreement between the model and experimental data (Huang et al., 2003); however, they showed that the use of a poroviscoelastic model improved fit quality. While the inclusion of viscoelasticity is certainly important for capturing the complete cartilage response it is left up to the reader to decide if this is a necessary design constraint.

The fit material parameters for bovine and porcine cartilage have similar relationships and their values agree with the existing literature (Soltz and Ateshian, 2000; Moore and Burris, 2015). In contrast, FiHy™ had a lower *E* and *ξ*, and higher *k*. Despite this, the modulus ratio (*ξ/E*) was »1 for all materials (bovine = 28, porcine = 100, FiHy™ = 20) enabling FiHy™ to support a majority of the applied load through poroelastic fluid pressure.

### 3.5 Osteochondral mechanics

Using the optimized parameters found for isolated cartilage (Table 4) as inputs to the fiber-reinforced model with a fully bonded contact did not predict the stress relaxation response of their osteochondral counterpart, Fig. 7 and Table 5. These findings are not surprising as it was previously established that having a bonded interface, such as subchondral bone or bone substitute, would drive higher FLS (Fig. 6). Optimizing the model fits for the fully bonded contact resulted in better curve-fits to the experimental data for both the porcine osteochondral specimen and FiHy™ + bone substitute, with fit values of 0.40 and 0.24. While the fiber-reinforced model with fully bonded contact is certainly capable of describing the first order response of the osteochondral specimen it could be further improved by considering additional physics, such as a non-rigid and nonlinearly elastic body to which it is bonded, transversely isotropic fiber distribution, and viscoelasticity.

Overall, the fiber-reinforced model provides a good first-order agreement with the experimental findings of both cartilage and FiHy™. Although there are more sophisticated constitutive models to describe the fiber behavior, and the *α* and *β* parameters could have been treated as optimizable parameters, we limited our model to account for the primary physics needed to demonstrate physiological poroelasticity. In terms of permeability, the fiber-reinforced model assumes constant isotropic permeability. In articular cartilage it has been shown that the permeability is anisotropic and strain-dependent (Soltz and Ateshian, 2000; Lai and Mow, 1980b). While model fits would certainly improve from higher order fiber models, directional permeability, and poro-viscoelasticity there remains limited experimental information to guide the appropriate selection of material parameters.

## 4 Closing remarks

The primary objective of this work was to identify the primary design requirements for developing a physiological poroelastic cartilagemimic. To this end we constructed two constitutive poroelastic models (poroelastic and fiber-reinforced poroelastic). The models were fit to bovine and porcine articular cartilage. While the poroelastic model was incapable of providing a predictive response, the fiber-reinforced model provided a good fit to experimental data. We then fit the fiber-reinforced model to a cartilage substitute known as FiHy™ (Moore et al., 2019). The model provided a first order description of the physics and suggests FiHy™ is at least partially driven by poroelasticity. Furthermore, we evaluated the effect of a bonded interface for cartilage and FiHy™ using both a theoretical and experimental approach. The results demonstrate that the bonded substrate enhances the fluid load support of the poroelastic body; however, the fit quality is reduced likely due to additional physics not considered in this first order model. Our future work aims to increase the fiber modulus and decrease the permeability of FiHy™ to produce a more cartilage-like response.

## Supplementary Material

Supplementary data to this article can be found online at https://doi.org/10.1016/j.jmbbm.2022.105528.

Supplementary Materials

## Figures and Tables

**Table 1:** Summary of the boundary conditions and rigid constraints implemented.

Body	Boundary Conditions
Top plate	Prescribed rigid displacement of –0.15 mm Fixed displacement in the x- and y-direction Fixed rotation in the x-, y- and z-direction Impermeable to fluid flow
Bottom plate	Fixed displacement in the x-, y- and z-direction Fixed rotation in the x-, y- and z-direction Impermeable to fluid flow
Poroelastic body	Fixed x-displacement in the yz-face of the body Fixed y-displacement in the xz-face of the body Zero fluid pressure for the curved surface

**Table 2:** Material parameters used in the poroelastic models.

Parameter	Value
Fluid volume fraction *φ^f^*	0.8
Permeability *k* (mm^4^/N-s)	0.001
Young's modulus *E* (MPa)	0.5
Poisson's ratio *ν*	0.1
Fiber modulus *ξ* (MPa)	4
*α*	0
*β*	2

**Table 3:** Geometry of poroelastic body in the fiber-reinforced model used for optimization. *The thickness of the poroelastic body is estimated to be 2 mm as only the overall thickness of FiHy™ + bone substrate is known (6.07 mm).

Measure	Bovine Cartilage	Porcine Cartilage	FiHy™	Porcine Osteochondral	FiHy + Bone Substitute
Thickness (mm)	2.130	2.160	2.030	2.400	2.000*
Radius (mm)	2.805	2.735	4.020	2.625	3.080

**Table 4:** Optimized parameters (Young’s modulus *E*, permeability constant *k*, and fiber modulus *ξ*) of the bovine cartilage specimen, porcine cartilage specimen, and FiHy™. The standard error of regression (S) from the fits are shown in the last row.

Parameter	Bovine Cartilage	Porcine Cartilage	FiHy™
*E* (MPa)	0.18	0.05	0.01
*k* (mm^4^/N-s)	0.04	0.10	3.00
*ξ* (MPa)	5.00	3.00	0.20
S (N)	0.12	0.42	0.48

**Table 5:** Optimized parameters (Young’s modulus *E*, permeability constant *k* and fiber modulus *ξ*) of the porcine osteochondral specimen and FiHy™ + bone substitute, and parameters obtained from their chondral counterpart. The standard error of regression (S) from the fits are shown in the last row.

Parameter	Porcine Osteochondral		FiHy™ + Bone Substitute	
	From Cartilage	Optimized	From FiHy™	Optimized
*E* (MPa)	0.05	0.05	0.01	0.10
*k* (mm^4^/N-s)	0.10	0.03	3.00	0.10
*ξ* (MPa)	3.00	3.00	0.20	1.00
S (N)	0.49	0.40	0.51	0.24

## Data Availability

Data will be made available on request.
