# Supplementary material for: Minimum design requirements for a poroelastic mimic of articular cartilage
Source: J Mech Behav Biomed Mater. Author manuscript; Available in PMC 2024 Jan 5. (PMC7615484; doi:10.1016/j.jmbbm.2022.105528)
Supplement: Supplementary Materials [file EMS175906-supplement-Supplementary_Materials.docx]

Title: Minimum design requirements for a poroelastic mimic of articular cartilage

Running Title: Minimum design for articular cartilage

Authors:

Wei S Tan^1^

Axel C Moore^1,2*^

Molly M Stevens^1*^

Author Affiliation:

^1^Department of Materials, Department of Bioengineering and Institute of Biomedical Engineering, Imperial College London, London, UK

^2^Department of Biomedical Engineering, University of Delaware, Newark, DE, USA

Author Contributions:

WST performed data curation, formal analysis, investigation, validation, and writing. ACM performed project conceptualization, formal analysis, investigation, methodology, supervision, validation, visualization, and writing. MMS performed funding acquisition, supervision, and writing.

*Corresponding Authors:

Axel C. Moore, PhD

Department of Biomedical Engineering

University of Delaware

Newark, Delaware 19713

USA

Tel: +1 302 319 7598

Email: axel@udel.edu

Molly M. Stevens, PhD

Department of Materials

Imperial College London

Royal School of Mines

London, SW7 2AZ

UK

Tel: +44 020 7594 6804

Email: m.stevens@imperial.ac.uk

**7.0 Supplemental Information and Extended Methods**

**7.1 Mesh Convergence**

The mesh density of the poroelastic body is determined by performing a mesh convergence study. This is to ensure that the model is producing a mathematically accurate solution without consuming too many computer resources to run the simulation. This is done by running the simulation with an increasing number of elements (finer mesh) and comparing the results (averaged effective fluid pressure). The mesh is refined in three directions: radial, tangential, and axial. To ensure that the averaged values of effective fluid pressure used for comparison are calculated at the same location in the poroelastic body, three areas of comparison are defined for each refinement direction, see **Fig S7.1A**. The mesh is considered optimized when the percentage difference between subsequent values of effective fluid pressure fall below 0.2%. The mesh is refined independently in each axis, and the combination of all three axes provides the optimized mesh that is used in this work.

**
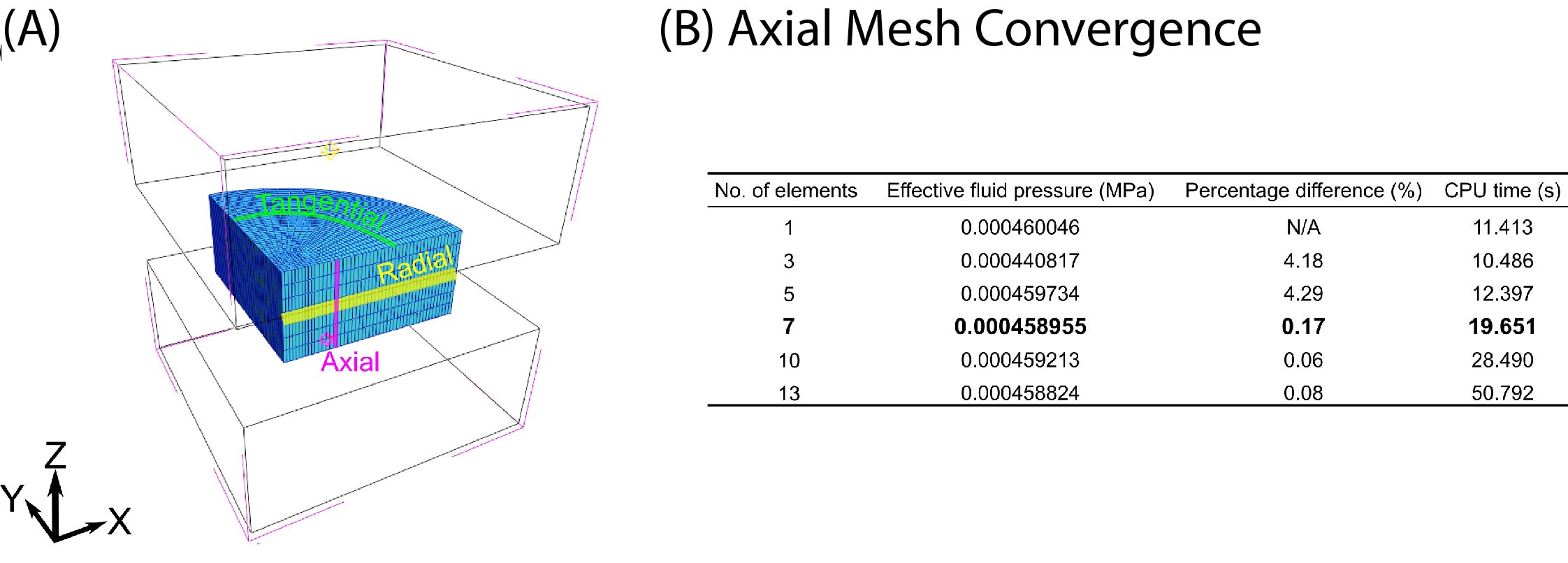
**

**Figure S7.1** (A) The optimized mesh for the poroelastic body. The top and bottom plate are hidden from view. The color bars indicate the locations where results are compared in the mesh refinement study: radial (yellow), tangential (green), and axial (purple). (B) Results of the mesh convergence study in the axial direction. NA denotes not applicable. Percentage difference refers to the percent difference between subsequent effective fluid pressure values while CPU time indicates the computational cost of the simulation. The optimized number of axial elements is 7.

**Table S7.1** Result of mesh convergence study in the axial direction.

| No. of elements | Effective fluid pressure / $\text{MPa}$ | Percentage difference / % | CPU time / $\text{s}$ |
| --- | --- | --- | --- |
| 1 | 0.000460046 | - | 11.413 |
| 3 | 0.000440817 | 4.18 | 10.486 |
| 5 | 0.000459734 | 4.29 | 12.397 |
| 7 | 0.000458955 | 0.17 | 19.651 |
| 10 | 0.000459213 | 0.06 | 28.490 |
| 13 | 0.000458824 | 0.08 | 50.792 |

**Table S7.2** Result of mesh convergence study in the radial direction.

| No. of elements | Effective fluid pressure / $\text{MPa}$ | Percentage difference / % | CPU time / $\text{s}$ |
| --- | --- | --- | --- |
| 11 | 0.00250031 | - | 10.581 |
| 15 | 0.00269710 | 7.87 | 5.896 |
| 20 | 0.00269896 | 0.07 | 8.952 |
| 30 | 0.00270459 | 0.21 | 12.397 |
| 40 | 0.00270501 | 0.02 | 19.048 |
| 50 | 0.00270555 | 0.02 | 25.572 |

**Table S7.3** Result of mesh convergence study in the tangential direction.

| No. of elements | Effective fluid pressure / $\text{MPa}$ | Percentage difference / % | CPU time / $\text{s}$ |
| --- | --- | --- | --- |
| 2 | 0.00255025 | - | 2.609 |
| 6 | 0.00279377 | 9.55 | 3.277 |
| 10 | 0.00281713 | 0.84 | 5.855 |
| 16 | 0.00282472 | 0.27 | 9.908 |
| 20 | 0.00282630 | 0.06 | 12.397 |
| 26 | 0.00282414 | 0.08 | 21.533 |
| 30 | 0.00282723 | 0.11 | 22.110 |

**7.2 Frictional verses rigid contact**


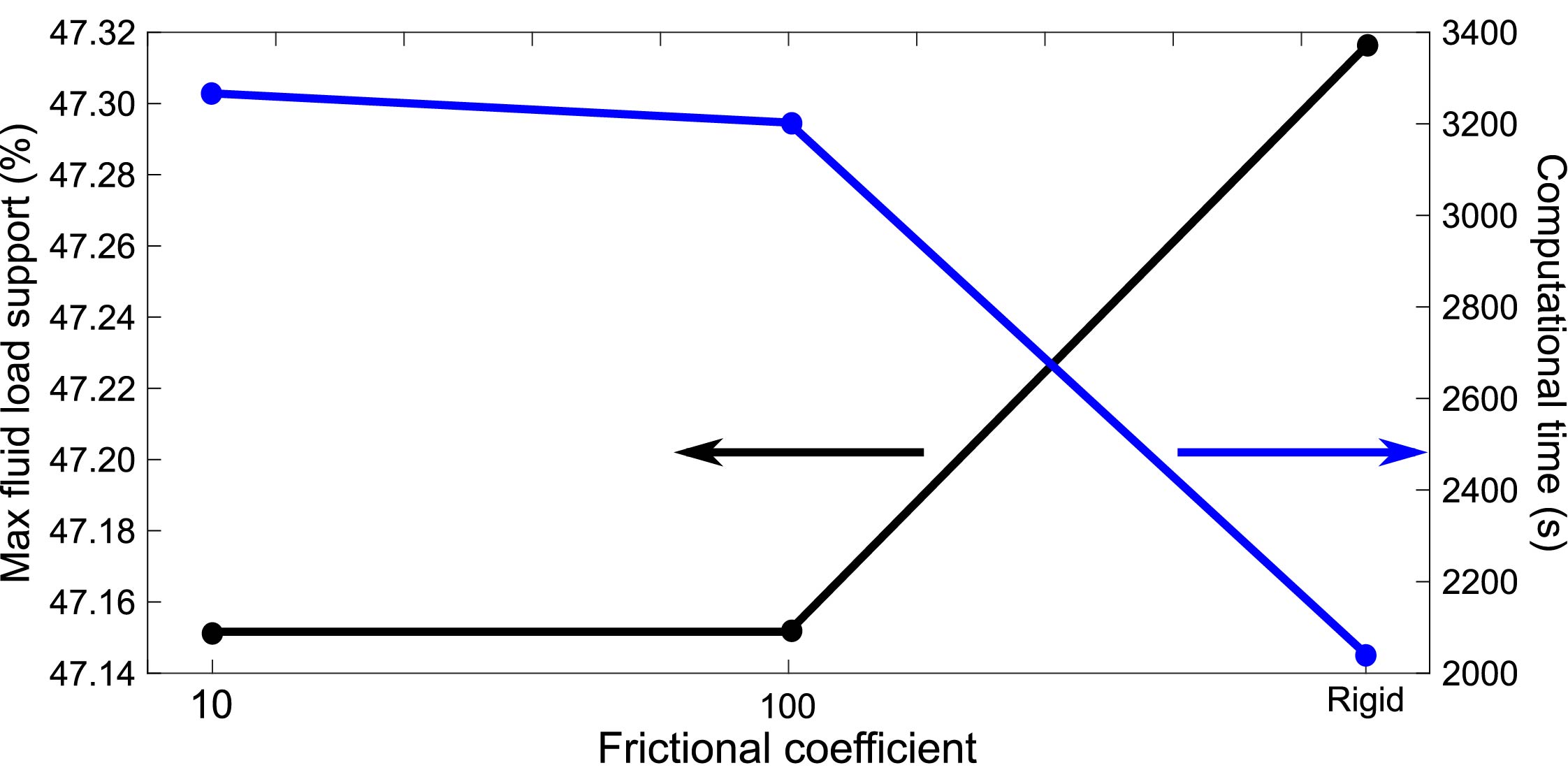


**Figure S7.2** Comparison of maximum FLS and computational time when the bottom contact is modeled as a sliding interface with a large friction coefficient of 10 and 100, and as a rigid contact.

The difference in maximum FLS between all three cases is negligible (~0.3%). However, the rigid contact reduced the computational time by 56.8% over the frictional interface. We use the rigid contact throughout this work given the good agreement and lower computational cost.

**7.3 Effect of poroelastic body thickness on fluid pressure and z-reaction force**

**
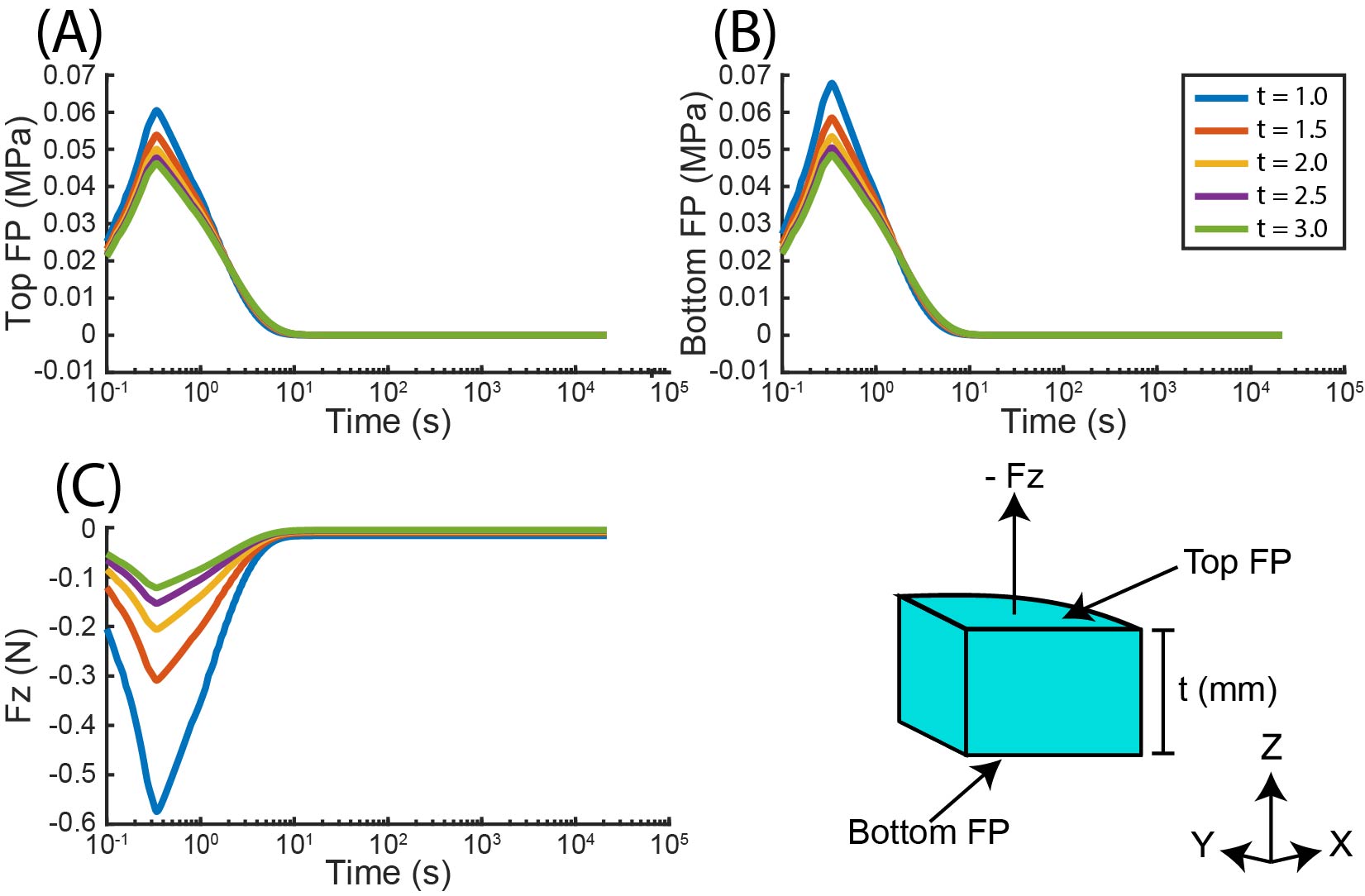
**

**Figure S7.3** Effect of the thickness of the poroelastic body on the fluid pressure evaluated on the (A) top surface and the (B) bottom surface, and on (C) the reaction force in the z-direction evaluated on the top plate (rigid body). These results are obtained from the fiber-reinforced model with a fully bonded contact.

As shown in **Fig S7.3**, the fluid pressure and reaction force of the system only vary with the thickness of the poroelastic body in the initial 100 s. However, it is observed that these variations decrease as the thickness increases. This finding demonstrates that quantifying poroelastic body thickness is an important parameter. The thickness of FiHy™ when it is attached to the bone substitute is estimated to be 2 mm since the thickness of FiHy™ only specimen does not exceed 2 mm.

**7.4 Effective fluid pressure distribution**

**

**

**Figure S7.4** Comparison of effective fluid pressure distribution at different time steps (time = 7, 12, 1933, 5263 s) for (A) poroelastic model (frictionless bottom contact), (B) fiber-reinforced model (frictionless bottom contact), and (C) poroelastic model (fully bonded contact)

It can be clearly seen from **Fig S7.4** that the fluid pressure within the poroelastic body in the fiber-reinforced model equilibrates most rapidly, followed by the poroelastic model with fully bonded contact and poroelastic model with frictionless bottom contact. The maximum fluid pressure in the poroelastic body (indicated by the color bar) also follows the same trend, where the highest maximum fluid pressure was observed in the fiber-reinforced model. These observations further support our conclusion that the confinement effect (from fiber reinforcement and fully bonded contact) drives higher FLS, and hence can be a potential means to enhance performance in cartilage implants.
